# Charged particle therapy for high-grade gliomas in adults: a systematic review

**DOI:** 10.1186/s13014-022-02187-z

**Published:** 2023-02-08

**Authors:** Yuhang Wang, Ruifeng Liu, Qiuning Zhang, Meng Dong, Dandan Wang, Junru Chen, Yuhong Ou, Hongtao Luo, Kehu Yang, Xiaohu Wang

**Affiliations:** 1 Institute of Modern Physics, Chinese Academy of Sciences, Lanzhou, China; 2 The First School of Clinical Medicine, Lanzhou University, Lanzhou, China; 3 Department of Postgraduate, University of Chinese Academy of Sciences, Beijing, China; 4 Heavy Ion Therapy Center, Lanzhou Heavy Ions Hospital, Lanzhou, China; 5 Evidence-Based Medicine Center, School of Basic Medical Sciences, Lanzhou University, Lanzhou, China

**Keywords:** Carbon-ion therapy, Proton therapy, High-grade gliomas, Systematic review

## Abstract

High-grade gliomas are the most common intracranial malignancies, and their current prognosis remains poor despite standard aggressive therapy. Charged particle beams have unique physical and biological properties, especially high relative biological effectiveness (RBE) of carbon ion beam might improve the clinical treatment outcomes of malignant gliomas. We systematically reviewed the safety, efficacy, and dosimetry of carbon-ion or proton radiotherapy to treat high-grade gliomas. The protocol is detailed in the online PROSPERO database, registration No. CRD42021258495. PubMed, EMBASE, Web of Science, and The Cochrane Library databases were collected for data analysis on charged particle radiotherapy for high-grade gliomas. Until July 2022, two independent reviewers extracted data based on inclusion and exclusion criteria. Eleven articles were eligible for further analysis. Overall survival rates were marginally higher in patients with the current standard of care than those receiving concurrent intensity-modulated radiotherapy plus temozolomide. The most common side effects of carbon-ion-related therapy were grade 1–2 (such as dermatitis, headache, and alopecia). Long-term toxicities (more than three to six months) usually present as radiation necrosis; however, toxicities higher than grade 3 were not observed. Similarly, dermatitis, headache, and alopecia are among the most common acute side effects of proton therapy treatment. Despite improvement in survival rates, the method of dose-escalation using proton boost is associated with severe brain necrosis which should not be clinically underestimated. Regarding dosimetry, two studies compared proton therapy and intensity‐modulated radiation therapy plans. Proton therapy plans aimed to minimize dose exposure to non-target tissues while maintaining target coverage. The use of charged-particle radiotherapy seems to be effective with acceptable adverse effects when used either alone or as a boost. The tendency of survival outcome shows that carbon ion boost is seemingly superior to proton boost. The proton beam could provide good target coverage, and it seems to reduce dose exposure to contralateral organs at risk significantly. This can potentially reduce the treatment-related dose- and volume-related side effects in long-term survivors, such as neurocognitive impairment. High-quality randomized control trials should be conducted in the future. Moreover, Systemic therapeutic options that can be paired with charged particles are necessary.

## Background

High-grade gliomas (HGGs) are the most common primary intracranial malignancies, accounting for approximately 47.1% of malignant brain tumors [1, 2]. According to the World Health Organization (WHO) classification, HGGs mainly consist of glioblastoma multiforme (GBM), anaplastic astrocytoma (AA), or oligodendroglioma [3]. The poor prognosis of aggressive treatment, including gross total resection, photon radiotherapy, chemotherapy, and(or) tumor treatment fields makes treatment challenging worldwide. Considering the features of highly infiltrative tumors and rapid progression, new treatment techniques need to be explored. Compared with X-rays, a high linear energy transfer beam has significant physical characteristics. The apparent decrease in the integral dosage is affected by a Bragg peak [4, 5]. Carbon ion beam has stronger biological effects than normal X-rays, and cell death caused by DNA double-strand breaks is far more difficult to repair. Some in vitro studies calculated the relative biological effectiveness of carbon ions in glioblastoma range between 3 and 5 [6, 7]. It is generally reported that the relative biological effectiveness of proton beams is 1.1 equivalent to that of photon [8]. Charged particles have been used to treat HGGs in the USA, Japan, Germany, and China. It is important to closely assess whether charged particles are effective and safe over current standard therapeutic options for treating HGGs. Therefore, we systematically reviewed the currently available data to thoroughly examine the clinical efficacy and safety of carbon ions and protons for treating HGGs and compare the results with those of conventional techniques.

## Materials and methods

### Search strategy

This systematic review was conducted according to the Preferred Reporting Items for Systematic Reviews and Meta-Analyses statement. All literature searches were conducted until July 2022, using the search tools of Embase, Cochrane Library, Web of Science, and PubMed databases with the search terms, “particle”, “charged particle”, “heavy-ion*”, OR “carbon ion”, OR “proton” AND “high-grade glioma” OR “glioblastoma” OR “anaplastic astrocytoma”. “particle”, “charged particle”, “heavy-ion*”, OR “carbon ion”, OR “proton” AND “Dosimetry” OR “Dosimetry study”. Only publications written in English were included. Additional studies were identified from the citation counts of conference abstracts, review articles, and reference lists. All references were screened to ensure that relevant studies were included.

### Inclusion and exclusion criteria

Studies were included if they met the following criteria: (a) clinical or retrospective studies reporting efficacy, adverse reaction, and/or dosimetry comparison in patients with newly diagnosed HGG with charged particle beam; (b) Trials enrolling adults; and (c) studies reporting tumors that had been pathologically diagnosed. Publications were excluded if they were (a) case reports on one or two patients; (b) letters, editorials, protocols, reviews, or meta-analyses; (c) duplicate publications; (d) cell and animal experimental studies; (e) lacking detailed data; (f) recurrent glioma; (g)prior brain radiation.

### Data extraction

The necessary data were extracted independently by two researchers, and the results were discussed with senior investigators if there was no discrepancy. Overall survival (OS), progression-free survival (PFS), treatment-related toxicity, target volume dose, and organ at risk dose were among the outcomes studied. For each article, the following data were obtained: first author, publication year, study period, the number of patients, institution, tumor site, tumor status, total treatment dose, and the median follow-up time.

### Quality assessment

Case series reports were evaluated using the case series report bias evaluation tool as shown in Table 1 with the answer yes, no, or ambiguous. The evaluation indicators were as follows: (a) inclusion and exclusion criteria; (b) clinical heterogeneity of patients, including disease severity, classification, duration, and onset time; (c) whether the main intervention measures (dose, administration, and course of treatment, etc.) were clearly described; (d) whether the measurement method of relevant outcome measures was reasonable; (e) whether the outcome measures were measured before and after the intervention; (f) whether the loss to follow-up and follow-up time were recorded; (g) whether adverse events related to clinical treatment were documented; and (h) whether the outcome measurer was blinded. The literature quality evaluation was independently completed by two members, respectively.

**Table 1:** Case series report quality evaluation form

Studies	a	b	c	d	e	f	g	h
Tsujii et al. [13]	No	Yes	Yes	Yes	Unapplicable	Yes	Yes	Unapplicable
Fitzek et al. [15]	No	Yes	Yes	Yes	Unapplicable	Yes	Yes	Unapplicable
Mizoe et al. [27]	No	Yes	Yes	Yes	Unapplicable	Yes	Yes	Unapplicable
Mizumoto et al. [17]	No	Yes	Yes	Yes	Unapplicable	Yes	Yes	Unapplicable
Adeberg et al. [14]	Yes	Yes	Yes	Yes	Unapplicable	Yes	Yes	Unapplicable
Vora et al. [11]	Yes	Yes	Yes	Yes	Unapplicable	Yes	Yes	Unapplicable
Kong et al. [25]	Yes	Yes	Yes	Yes	Unapplicable	Yes	Yes	Unapplicable
Brown et al. [12]	No	Not clear	Yes	Yes	Unapplicable	Yes	Yes	Unapplicable

a–h: Case series reports were evaluated using the case series report bias evaluation tool item (refer to Quality assessment paragraph)

## Results

### Study characteristics

**Fig. 1:**
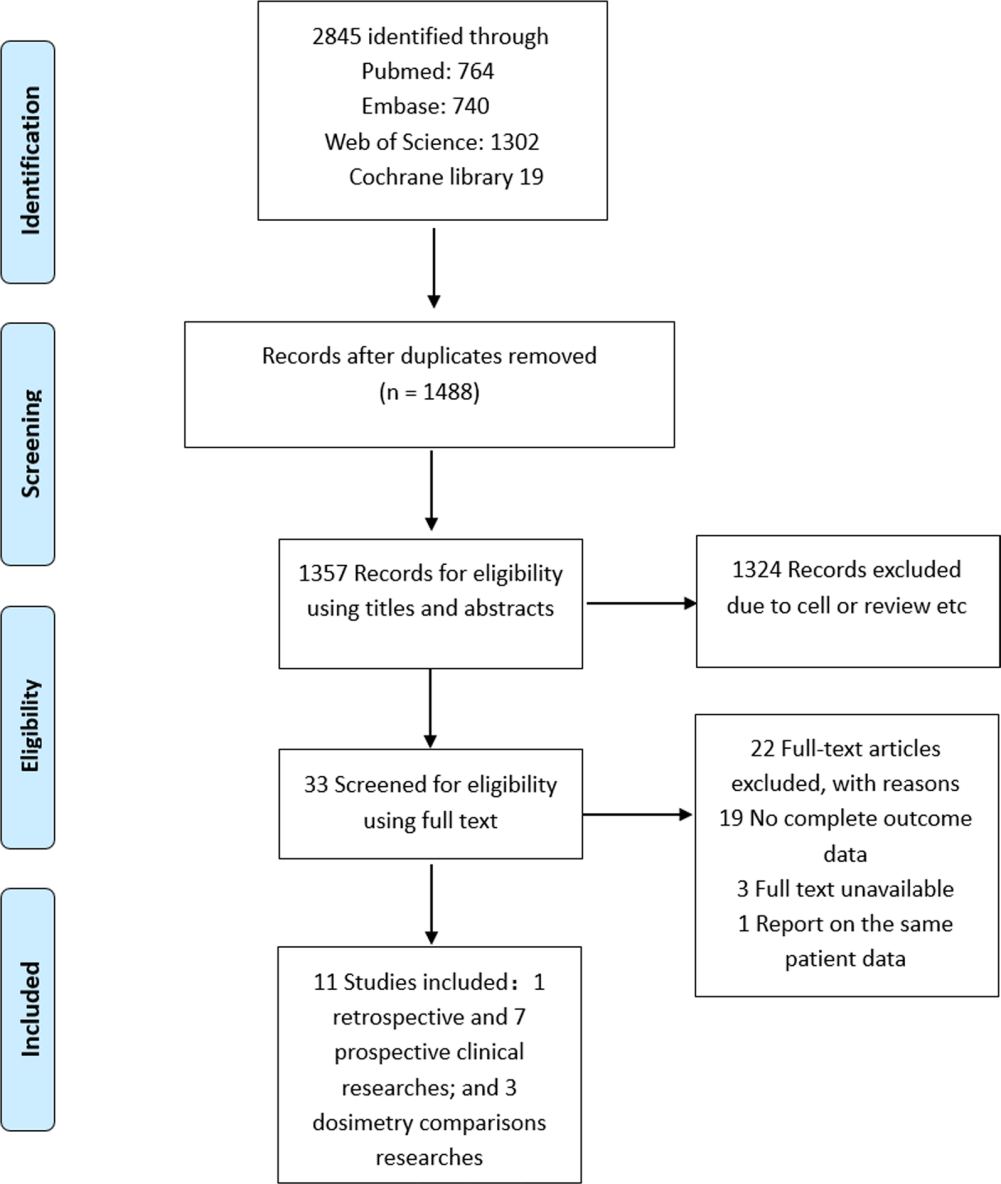
PRISMA flow diagram

During the duplication removal and abstract screening process, 1357 of the original 2845 studies classified by four databases were removed (refer to Fig. 1). According to the applicable exclusion criterion, 33 full texts were screened further, leaving 11 relevant studies for data extraction. Eight clinical trials and three dosimetry comparability studies were conducted. Eight clinical studies were chosen (one retrospective study, seven prospective studies) including two carbon-ion-related treatments, and six protons and/or photon-based combinations. A total of 350 patients were drawn from eight cohorts, with case numbers ranging from 13 to 67, and the median follow-up times ranging from 14.3 to 48.7 months. All 350 patients were diagnosed with HGG (WHO grade III–IV), and the pathological types, including glioblastoma and AA. Two prospective studies associated with carbon-ion therapy were undertaken in Japan and China, while the rest of the research was conducted in two countries (USA, Germany). The general characteristics are shown in detail in Table 2. Despite some inconsistencies in the definitions of both regions of interest (for example, the healthy brain tissue) and relevant dosimetry indices (for example, inhomogeneity coefficients and conformity indices), which hindered data pooling, most dosimetry studies reported a statistical analysis providing quantitative support for the results. The ongoing clinical trials are reported in a Table 5.

**Table 2:** Baseline characteristics of included studies

Studies	Country	Study design	Time range	Surgery			Radiation modality	Chemotherapy	No. of patients	Median Follow-up(mo)	Charged particle technique summary
				GRS	SUB	BIO					
Tsujii et al. [13]	Japan (TSU)	Prospective	1983–1990	–	–	–	Proton ± Photon	No	13	–	NG
Fitzek et al. [15]	USA (MGH)	Prospective	1992–1996	9	13	1	Proton + Photon	NG	23	33	NG
Mizoe et al. [27]	Japan (NIRS)	Prospective	1994–2002	8	8	32	Photon + Carbon ion	ACNU	48	23	NG
Mizoe et al. [27]	Japan (TSU)	Prospective	2001–2009	0	15	8	Proton + Photon	TMZ or ACNU	23	–	Double-scattering proton therapy
Adeberg et al. [14]	Germany (HIT)	Retrospective	2011–2015	–	–	13[10]^b^	Proton + Photon	TMZ^a^	132	15	Proton beam: active raster-scan system
Vora et al. [11]	USA (Multi-institution)	Prospective	2009–2017	34	18	11	Proton alone	TMZ^a^	63	15	NG
Kong et al. [25]	China (SPHIC)	Prospective	2015–2018	17	22	11	Proton + Carbon ion	TMZ^a^	50	14.3	PRT/CIRT: 2–3 beams; Pencil-beam scanning system
Brown et al. [12]	USA (UT MDA)	Prospective	2013–2016	19(20)^b^	5[17]	2(4)	Proton vs. IMRT	TMZ^a^	67	48.7	IMPT: Multi-or single-field optimization; Passive or active-scatter technique

*TSU* University of Tsukuba; *MGH* Massachusetts General Hospital; *NIRS* National Institute of Radiological Sciences; *HIT* Heidelberg Ion-Beam Therapy Center; *SPHIC* Shanghai Proton and Heavy Ion Center; *UT MDA* The University of Texas, MD Anderson Center; *GRS* Gross total resection, *SUB* Subtotal resection, *BIO* Biopsy; *IMRT* intensity modulated radiation therapy; *ACNU* Nimustine hydrochloride; *TMZ* Temozolomide; *NG* Not given concretely

*PRT* proton radiotherapy, *CIRT* carbon ion radiotherapy, *IMPT* intensity modulated proton therapy

^a^TMZ guided by Stupp et al.

^b^Surgery population of IMRT group

### Treatment and clinical survival results

As shown in Tables 2 and 3, the eight selected articles included one retrospective study, six prospective phases I/II mono-institutional studies, and one multi-institutional collaborative registry. Overall, 350 patients with newly diagnosed HGGs were treated. The median follow-up ranged between 14.3 and 48.7 months. Two clinical studies regarding the treatment center of Japan and China conducted carbon-ion-related therapy. These two trials were both prospective and single-center trials, evaluating the outcome of carbon ion or mixed with proton or photon therapy after some surgical procedure. In the National Institute of Radiological Sciences, patients were treated using photon therapy and chemotherapy followed by carbon ion therapy. Photon therapy (1.8 to 2.0 Gy per fraction) was administered five days a week with a total of 50.0 Gy. Nimustine hydrochloride (ACNU) was administrated at a dose of 100 mg/m^2^ on the first and fourth or fifth weeks of photon therapy. After radio-chemotherapy for each patient, carbon ion radiation was administered four days per week for over two weeks. In a dose-escalation study from 16.8 Gray equivalent (GyE) to 24.8 GyE in every 10% increase, the median OS of AA patients was 35 months while those with GBM was 17 months. The median PFS and OS of patients with GBM were 4 and 7 months for the low-dose group (16.8GyE), 7 and 19 months for the middle-dose group (18.4–22.4GyE), and 14 and 26 months for the high-dose group (24.8GyE), respectively. In a clinical trial by Kong et al., 50 patients were treated with a proton or proton plus carbon ion boost. The proton therapy protocol was 60.0GyE in 30 fractions, with three dose-escalation schemes employing carbon ions in addition to chemotherapy (refer to Table 3). The 12-month and 18-month OS rates were 87.8% and 72.8%, respectively. The 12-month and 18-month OS rates of GBM were 77.4% and 61%, respectively, and those of AA were 100%. The efficacy of these two carbon-ion-related radiations had an advantage over current standardized protocols from Stupp et al. [9, 10].

**Table 3:** Main radiotherapy results of included studies

Studies (year)	Median age (range)	Median dose in Gy (RBE)	Fraction (Fx)	Gy (RBE)/Fx	OS% (year)/median OS	PFS% (year)/ median PFS
Tsujii et al. [13]	AA:42.6 GBM:60.4	Proton alone (GBM):66.8 Gy; Photon + Proton_boost_ (AA):86.8 Gy	Proton alone:23 to 25Fx Photon:16 to 27FxProton_boost_:15 to 17Fx	Proton alone:2.5 to 3.5 Gy Photon + Proton_boost_:1.8 Gy	mOS(AA/GBM/AA + GBM):25,13,20mo	–
Fitzek et al. [15]	51(21–68)	Photon + Proton_boost_:total dose 93.5CGE(92.05 to 94.2 CGE)	–	Photon:1.8 Gy; Proton_boost_: 1.92 CGE	OS%:78%(1y), 34% (2y), 18%(3y); mOS 20mo	–
Mizoe et al. [27]	53(18–78)	Photon 50 Gy + CIRT_boost_:16.8 to 24.8GyE (16.8,18.4,20,22.4,24.8)	Photon:25Fx + CIRT_boost_:8Fx	Photon:2 Gy; CIRT_boost_:2.1 to 3.1 GyE	mOS: AA 35mo; GBM 17mo; mOS:GBM(high-dose, middle-dose, low-dose):26mo,19mo, 7mo	mPFS: GBM (high-dose, middle-dose, low-dose): 4mo, 7mo, 14mo
Mizumoto et al.[17]	56(31–76)	Photon 50.4 Gy; after 6 h proton_boost_ 46.2GyE	Photon:28 Fx; after 6 h proton_boost_:28 Fx	Photon:1.8 Gy; after 6 h proton:1.65GyE	OS%:78%(1y),43%(2y); mOS 21mo	PFS%(1,2y):36%,13% mPFS:9mo
Adeberg et al. [14]	Photon:57.9 (21.6–77.9); Photon + Proton boost:57.9 (20.0–77.0)	Photon:50.0(50.0 to 50.4) Gy/25(25 to 28) Fx; Photon + Proton_boost_:50 Gy + 10GyE/5Fx	Photon:25–28Fx; Proton_boost_:5Fx	Photon:1.8–2 Gy; Proton boost:2GyE	mOS: Photon:20.9mo; Photon + proton_boost_:19.1mo	mPFS: Photon:7.2mo; Photon + proton_boost_:8.8mo
Vora et al. [11]	56	Proton: 59.4 GyE (range 40–66 GyE)	15–33Fx	–	mOS 18.3 mo OS%:39%(2y); mOS GBM 17mo	–
Kong et al. (25)	54.5(22–76)	Proton:60 GyE; Proton:50 GyE + CIRT_boost_:10 to12 GyE; Proton:60 GyE + CIRT_boost_: 9 to12GyE; Proton:34 GyE + CIRT_boost_:9 GyE	Proton:30Fx; Proton:25Fx + CIRT_boost_ 4 to 5Fx; Proton 30 Fx + CIRT_boost_ 3Fx; Proton:10 Fx + CIRT _boost_:3Fx	Proton 2 GyE; Proton, 2 GyE + CIRT_boost_ 2–3GyE; Proton 2 GyE + CIRT_boost_ 3–4 GyE; Proton 3–4 GyE + CIRT_boost_ 3 GyE	OS%(1y,1.5y):87.8%,72.8%; OS%(1y,1.5y): AA 100%; GBM:77.4%, 61%	PFS%(1y,1.5y):74.2%,59.8%; PFS%(1y,1.5y): AA 100%; GBM:61.3%, 42.7%
Brown et al. [12]	GBM: IMRT:53 (26,82); PT:54.5 (33,72)	PTV1^a^:50 Gy PTV2^a^:60 Gy	25Fx	2 Gy/2GyE	mOS^a^: IMRT 21.2mo Proton 24.5mo	mPFS^a^: IMRT 8.9mo Proton 6.6 mo

*OS* overall survival; *GBM* glioblastoma; *AA* anaplastic astrocytoma; *CGE* Cobalt Gray Equivalent; *mo* month; *y*: year; *RBE* relative biological effectiveness; *CIRT* Carbon ion radiotherapy

^a^PTV1 (Plan tumor volume1): CTV1 (Clinical tumor volume1) + 3–5 mm; PTV2:GTV (Gross tumor volume) + 3–5 mm; CTV include GTV + 2 cm anatomically constrained margin; *Fx* fractions

^a^No significant difference in mOS and mPFS (IMRT vs. Proton Therapy)

The remaining six studies were associated with proton and/or photon therapy. In the study by Vora et al. [11], using prospective, multi-institutional clinical trials among HGGs were conducted using therapeutic surgery and temozolomide-based chemotherapy followed by proton radiation alone. The median total dose delivered was 59.4 GyE (range 40 to 66 GyE), which was administered over 15–33 fractions. The median OS for all patients was 18.3 months with 2 years OS of 39.0%, and the median OS for GBM was 17 months. The next study contributed to this systematic review from Brown et al. [12]; the enrolled patients were randomized to proton therapy or intensity-modulated radiation (IMRT) groups. Similarly, surgery and chemotherapy were performed in preparation for subsequent radiotherapy. The simultaneous integrated boost technique treated both PTV50 and PTV60 to 50 Gy and 60 Gy in 30 fractions. There was no difference between the proton therapy and IMRT in median PFS (6.6 months vs.8.9 months) or OS (24.5 months vs.21.2 months).

At different Japanese, German, and USA institutes, the University of Tsukuba explored novel approaches for photon and proton boost therapies. In the study by Tsujii et al. [13], five patients were treated with proton therapy alone with a median 66.8 Gy, while eight patients were treated with photon therapy followed by proton boosts for a total mean 86.8 Gy. The median OS for AA, GBM, and AA + GBM were 25, 13, and 20 months, respectively. Mizumoto et al. [14] reported that patients who underwent photon therapy (total dose 50.4 Gy in 28 fractions) followed by hyper-fractionated boost proton therapy (total dose 46.2GyE in 28 fractions) plus nimustine hydrochloride (80 mg/m^2^ in the first and fourth weeks) or temozolomide(75 mg/m^2^, daily) had 1- and 2-year OS rates of 78% and 43%, respectively and median OS of 21.0 months. The treatment plan of studies by Adeberg et al. [14] also included treating the patients with photons (range 50 to 50.4 Gy) and proton boost (total dose 10GyE); the median OS for photon alone and photon plus proton boost was 20.9 and 19.1 months, respectively. Fitzek et al. [15] reported that 23 GBM patients underwent surgery followed by accelerated fractionated photon therapy (total dose 55–65 Gy) and proton therapy applied as a boost (dose escalation to 90 cobalt gray equivalent, CGE) in the United States. The prescription doses for the three target volumes in this prospective phase II trial are, respectively, volume 1, 90 CGE; volume 2, 64.8 CGE; and volume 3, 50.4 CGE (proton doses plus x-ray irradiation doses). The median total dose was 93.5 CGE with 92.05 to 94.2 CGE. The OS rates at one, two, and three years were 78%, 34%, and 18%, respectively. The median survival time was 20 months.

### Patterns of failure

The patterns of failure (mainly tumor recurrence) are reported by Fitzek et al. and Mizumoto et al. [15, 16]. Pathological samples were available in 15 (65%) of 23 patients from the dose-escalation plan. On the contrary, nine patients were found to have recurrent tumors and radiation necrosis. Further analysis on three patients by autopsy demonstrated that recurrent tumors were outside the high-dose volume (90CGE). The Kaplan–Meier analysis showed that the median OS of the patients with necrosis was 29 months compared with 16 months in the recurrent patients (*p* = 0.01). Another study reported that MRI examined all 23 patients treated by hyper-fractionated proton boost. In another study of 23 patients who received hyper-fractionated proton boost, changes in control MRI occurred in 20 cases. Six (75%) of 8 patients within who received 96.6GyE had brain necrosis without tumor recurrence within CTV1.

### Toxicity

Almost all patients completed the treatment without interruption or discontinuation. The study examined acute toxicities within 3–6 months of treatment completion. Two studies have reported adverse reactions in patients with HGGs treated with carbon ion boost. No Grade 3 or severe toxicities were reported. These cases reported some acute symptoms following carbon ion boost, such as dermatitis, alopecia, headaches, and late side effects relating to brain necrosis in patients with Grade 1–2 (according to CTCAE or RTOG/EORTC). All of the data are shown in Table 4. The side effects of the photon with proton boost from Adeberg et al. [14] showed that toxicity of Grade 1–2 was observed in 6 patients receiving bimodality radiotherapy and no Grade 3 or higher-grade acute reactions. And late side effects were not reported. Dose escalation using protons as boost to the photon therapy offers a high total dose. The common acute adverse effects (Grade1-2) were alopecia/dermatitis, nausea, or headache, and one patient had a severe acute reaction. Still, brain necrosis was a very common late toxicity (23% of patients with radiation necrosis by Mizumoto et al. [17] and all patients in the study of Fitzek et al. [15]). Similarly, the most common acute adverse effects during treatment with proton therapy were dermatitis, headaches, and alopecia, while radiation necrosis has not yet occurred [11]. Brown et al. [12] reported that proton therapy significantly reduced the dose of normal tissues. When adverse event occurrences are compared for each patient, the average number of Grade 2 or higher toxicities is considerably higher in patients who received IMRT than in those who underwent proton therapy.

**Table 4:** Acute and late adverse effects

Study	Patients	Radiation modality	Evaluation criterion	Severe acute or late toxicity (≥ G3)	Common acute toxicity (G1–2) n (%)	Common late toxicity(G1–2) n (%)
Mizoe et al. [27]	48	Photon (50 Gy in 25 fractions) + Carbon ion boost (highest dose 24.8GyE in 8 fractions)	RTOG/EORTC	None	Alopecia 36 (75%) caused by XRT	Brain necrosis 4(8.3%)
Kong et al. [25]	50	Proton (34–60GyE in 10–30 fractions) + Carbon ion boost (2–4GyE in 3–5 fractions)	CTCAE v.4.03	None	Alopecia/Dermatitis 29 (76%)	Brain necrosis 11(22%)
Adeberg et al. [14]	66	Photon (50 Gy in 25 fractions) + Proton boost (10GyE in 5 fractions)	CTCAE v.4.03	None	Increased intracranial pressure 4(6%), Seizure 1(2%), Decreased fine motor skills 1(2%)	–
Mizumoto et al. [17]	23	Photon (50.4 Gy in 28 fractions) + Proton boost (46.2GyE in 28 fractions)	CTCAE v.3.0 RTOG/EORTC	None	Nausea or Headache 5(21.7%), Alopecia/Dermatitis 23(100%)	Brain necrosis 6(26%)
Fitzek et al. [15]	23	Photon + Proton boost (median total dose 93.5CGE)	RTOG/EORTC	Cerebral edema 1(4.3%)	Nausea 8(34%), Dizziness 2(8.6%), Headache 2(8.6%), blurred vision 1(4.3%), Alopecia /Dermatitis 23 (100%)	Brain necrosis 23(100%)
Tsujii et al. [13]	13	Photon + Proton boost	–	–	–	–
Brown et al. [12]	28(proton) 39(IMRT)	Proton (60GyE in 30 fractions) vs. IMRT (60 Gy in 30 fractions)	CTCAE v.4.03	Proton: Hydrocephalus 1(3.6%) IMRT: Headache 1(2.6%)	Proton: Alopecia 19(67.9%), Fatigue 17(60.7%), Headache 8(28.6%); IMRT: Alopecia 29(74.4%), Fatigue 26(67%), Headache 19(48.7%)	–
Vora et al. [11]	63	Proton (40–66GyE in 15–33 fractions)	CTCAE v.4.03	Headache 1 (1.6%), Fatigue 1 (1.6%), Thrombocytopenia 1 (1.6%)	Alopecia/Dermatitis 63 (100%), Headache 63 (100%), ataxia 63(100%), confusion63(100%), insomnia63(100%)	/

*CTCAE* Common Terminology Criteria for Adverse Events version 3.0/4.03 (CTCAE v3.0/4.03); *RTOG/EORTC* Radiation Therapy Oncology Group/European Organization for Research and Treatment of Cancer; *G1–2* Grade1–2 *G3* Grade3; /: NOT GIVEN

### Comparison of dosimetry plans

Three available papers were related to dosimetry studies, comparing 3D-CRT, intensity‐modulated radiation therapy (IMRT), and proton therapy, as well as different IMRT techniques, such as volumetric modulated arc therapy (VMAT) versus intensity-modulated proton therapy. The standard photon therapy radiation plan was prescribed at 60 Gy in 30 fractions, while the patients received intensity-modulated proton therapy (IMPT) with 60GyE in 2.0GyE fractions. Holm et al. explored the dose-escalation plan for all three techniques (refer to Table 6).

#### Target dosimetry evaluation

Adeberg et al. [18] showed that clinical target volume(CTV)coverage was comparable among three distinct techniques; V_90%_ and V_95%_ were observed with no difference. The homogeneity index (HI = D_5_ − D_95_/D_p_) was higher in the proton and VMAT plans than in the 3D-CRT plans. The minimum dose to the CTV (Dmin) in the proton plans was significantly higher than in the photon plans (*p*-value < 0.05). The PTV coverage, absorbed dose standard deviation for PTV, was the only target parameter illustrated by Rosenschöld et al. [19] research. The PTV coverage was significantly higher for IMPT (*p* < 0.0001) than VMAT. Holm et al. [20] explored dose escalation of three modalities using two parameters (R30 and ODV refer to Table 5 annotation) to evaluate dose homogeneity. The median ODV was reduced by 4 percentage points (*p* < 0.05) and 48 percentage points (*p* < 0.05) for the VMAT and IMPT, respectively. The IMPT significantly reduced the median R30 (45% vs. 35%, *p* < 0.05).

**Table 5:** The Ongoing clinical trials with Carbon ion or Proton therapy. Source: (Clinicaltrials.gov) or (www.ptcog.ch), updated November 2022

Study title	ID	Sponsor	Pathology	Phase	Arms
Carbon ion radiotherapy for primary Glioblastoma (CLEOPATRA)	NCT01165671	University Hospital Heidelberg	Glioblastoma	II	Photon(48–52 Gy) with concurrent TMZ plus Carbon ion boost (18GyE in 6 fractions) or Proton boost (10GyE in 5 fractions)
Proton and heavy ion beam vs. photon beam radiation for newly diagnosed Glioblastoma	NCT04536649	Shanghai Proton and Heavy Ion Center	Glioblastoma Anaplastic astrocytoma	III	Arm A: Standard-dose photon therapy (60 Gy in 30 fractions for high-risk area) plus TMZ; Arm B: Standard-dose Proton therapy (60GyE in 30 fractions for high-risk area) plus TMZ; Arm C: Carbon-ion boost (15GyE in 3 fractions for residual lesion) plus Standard-dose Proton therapy (60 Gy in 30 fractions for high-risk area) plus TMZ
Dose-escalated photon or proton therapy vs. standard-dose Photon therapy and TMZ for newly diagnosed Glioblastoma	NCT02179086	NRG Oncology	Glioblastoma	II	Arm A (control): Standard-dose photon therapy (60 Gy in 30 fractions for high-risk area) plus TMZ; Arm B (experiment): Dose-escalated Photon therapy Arm C (experiment): Dose-escalated Proton therapy
Proton Radiation Therapy for Gliomas	NCT01358058	Massachusetts General Hospital	WHO Grade 3 Glioma with IDH1 mutation or 1p/19q codeletion	Not applicable	Single arm study delivering fractionated proton therapy over 6 week (54–59.4 Gy (RBE) at 1.8 Gy (RBE))
Proton or IMRT in Preserving Brain Function in Patients with IDH Mutant Grade II or III Glioma	NCT03180502	NRG Oncology	WHO Grade 3 Glioma with IDH1/2 mutation or 1p/19q codeletion	II	Arm A (control): Standard-dose Photon therapy (60 Gy in 30 fractions for high-risk area) plus TMZ; Arm B (experiment): Standard-dose Proton therapy (60 Gy in 30 fractions for high-risk area) plus TMZ;
Proton Radiotherapy for Primary Central Nervous System Tumors in Adults	NCT02797366	Uppsala University	WHO Grade 3 Glioma with IDH1 mutation or 1p/19q codeletion	Not applicable	Single arm study delivering Proton radiation therapy daily (Monday through Friday) for 4–8 weeks
Comparison of Proton and Photon Radiotherapy of Brain Tumors	NCT02824731	Technische Universität Dresden	High-grade Glioma	Not applicable	Single arm study delivering fractionated proton therapy (54–60 Gy (RBE) at 2.0 Gy (RBE))

*IMRT* intensity modulated radiation therapy; *TMZ* temozolomide; *GyE* Gray equivalent

#### Dosimetry assessment of OARs

Three papers provide specific figures on OARs: Adeberg et al. [18] reported a significant reduction in the Dmean to the contralateral optic nerve (ON) and brainstem in IMPT compared with VMAT (− 66.8 and − 28.1%, respectively) or 3D-CRT (− 67.6 and − 67.7%, respectively). As described above, the integral dose was reduced when observed on the contralateral optic nerve and brainstem. IMPT reduced the Dmean and integral dosage to the pituitary gland by 52.5% and 65.0%, respectively, along the hypothalamic-pituitary axis. The Dmean and ID to the infratentorial brain could be reduced by 77.0% and 81.7%, respectively, when using IMPT over VMAT and by 91.6 and 81.7%, using 3D-CRT. Adeberg et al. [18] also demonstrated that sparing of the contralateral subventricular zone (SVZ) and hippocampus was significant for Dmean and integral dose when using proton therapy. According to Rosenschöld et al. [19] the Dmean to the whole brain was significantly lower for IMPT than VMAT (*p* < 0.0001), while the Dmax to the chiasm and brainstem were comparable for all techniques. Holm et al. [20] compared dose-escalation plans and the standard plan in the Dmean to the OAR. For IMPT, the sparing of all the organs at risk was much better than the standard plan when noticed. IMPT significantly reduced the danger of radio-necrosis by estimating the ratio of the dose-volume histograms of the brain.

## Discussion

This systematic review aimed to determine the efficacy and safety of charged-particle therapy in adult patients with HGGs. However, few articles are available that are relevant to the aim of this research. The application of charged-particle therapy for treating HGG was now basically in clinical trials. Moreover, these accessible researches, in general, contain inadequacies, such as small sample sizes, a heterogeneous body of research, and obsolete diagnosis or treatment measures. Over the past decade, there has been a consensus on the management of gliomas. For newly diagnosed HGGs, the standard treatment approach includes maximally safe surgery, concurrent TMZ-based radio-chemotherapy (75 mg/m^2^ for 42 days), adjuvant chemotherapy (150–200 mg/m^2^ in a 5/28 schedule) for 6–12 cycles, further combined with tumor treatment fields (TTF) for GBM but also for AA in some countries. The radiation dose prescription is up to 60.0 Gy in 2.0 Gy fractions that include the contrast-enhancing area [9, 10, 21]. Even though the median OS and median PFS for radiation plus temozolomide for GBM were 14.6 and 6.9 months, the outcome of survival is still unfavorable [22]. Strategies regarding dose-escalated radiotherapy, hypo-fractionated or hyper-fractioned trial, and stereotactic radiosurgery boost were conducted [23, 24]. However, these active attempts do not seem to fundamentally solve the problem of tumor control.

Up till now, the carbon-ion beams have been applied in clinical practice as boosting technique after proton or photon therapy. Kong et al. [25] reported that the modality of particle therapy plus concurrent temozolomide was carried out to manage HGG for the first time. The 18 months OS and PFS rates with charged particle therapy plus temozolomide were 72.8% and 59.8%, respectively, and 29.4% and 18.4% (18 months) with photon-based chemo-radiotherapy by Stupp et al. [10]. The above results indicate that charged particle therapy (proton plus carbon ion boost) appears to be more effective than traditional photon therapy. However, the median follow-up of 14.3 months is too short to be accurate, and the subsequent results should be considered constantly. The pattern of treatment failure in HGGs is mainly local recurrence, even if maximal surgery and high-dose radiotherapy are completed. The majority of recurrence sites are related to photon therapy CTV [26]. Dose escalation may solve the problem if the brain tissue is spared from high-dose radiation. In the Shanghai Proton and Heavy Ion Center (SPHIC) study, most patients who had residual disease after surgery were treated with high-dose proton therapy followed by a carbon-ion boost; this strategy seems effective in the short term. In another Phase I/II carbon ion dose-escalation trial, Mizoe et al. [27] observed the median OS of AA patients was 35 months, and that of GBM patients was 17 months (*p* = 0.0035). The median PFS of GBM was 4 months for the low-dose group and 7 and 14 months for the middle-dose and high-dose groups, respectively. The univariate and multivariate analyses of prognostic factors of GBM patients showed that high-dose carbon ion groups had significantly better OS than middle or low-dose groups. Although the number of cases in this series was limited, the median overall survival of patients with WHO grade III and IV gliomas were 35 and 17 months, respectively, which appears to be favorable. To avoid severe and unacceptable radiation toxicity to normal brain tissue, the T2 high-signal area of MRI was treated with photons rather than carbon ions, whose effect was unknown. (Because there had been no research with a clinical application of carbon ions for the treatment of human brain tumors before this study).

With the two main advantages of the heavy particle beam, the adverse effects simultaneously become concerning. Even with high-dose carbon-ion irradiation, triple modalities paired with photon, ACNU, and Carbon ion boost are safe for normal brain tissue. Nonhematologic side effects were uncommon, and there were no Grade III or IV reactions in the brain (the ACNU chemotherapy regime probably caused hematologic toxicities). Because of severe hematological toxicity in patients, ACNU has not been recommended as a chemotherapy guideline. Furthermore, the ineffectiveness of ACNU is most likely due to dosage limitations and the blood–brain barrier [27, 28]. The observation, in particular, was that brain necrosis in the target region using magnetic resonance imaging was only restricted to perilesional necrosis. The symptoms of headache and fatigue in this study are prevalent side effects [29, 30]. According to this Phase I/II trial, carbon ion radiation is safe for normal brain tissues. It would provide a basis for treating gliomas with carbon ion beams. The next phase clinical trial consisting of a carbon ion beam will continue to be conducted. However, a slightly different result from Kong et al. [25] showed 11 patients experienced grade I–II late side effects of radiation-induced brain necrosis after the completion of particle therapy. The late effect of CIRT on brain tissue is unknown due to the short follow-up period. It is difficult to distinguish between brain necrosis and pseudoprogression after radiation in the short term [31–33]. The issue of CIRT-induced brain necrosis cannot be overlooked. Hasegawa et al. [34] reported that severe late toxicities in the normal brain occurred after irradiation with CIRT alone (55.2 GyE in 24 fractions). High biological effectiveness results in significant cell-killing outcomes, however, such properties might cause severe irreversible organ dysfunction. Alopecia, along with other acute toxicities such as dermatitis, is a common and reversible symptom. The lack of severe acute side-effects has shown the potential safety of the carbon ion beam. These findings are significantly important for carbon ion beam to corroborate the physical advantages, which could reduce side effects compared to conventional radiation. The neurocognitive function should also be better preserved, in particular in patients without MRI changes like brain necrosis and cerebral edema. But these main complications of intracranial irradiation are not reported after using carbon ion therapy. Bevacizumab, steroid agents, or mannitol could alleviate brain edema generated by radiotherapy [35]. Evaluating neurocognitive function and quality of life (QoL) with ion-radiotherapy might be a direction of research. Still, the poor prognosis of HGGs seemingly impedes long-term follow-up.

Protons have physical features similar to carbon ion beams; however, their biological efficiency is inferior to that of heavy particles, which are roughly 1.1–1.2 equivalent to photons [8, 36, 37]. The role of proton therapy remains controversial due to its efficacy and high cost. Several institutes conducted a series of investigations on clinical trials involving protons, either alone or in combination with photon techniques. Vora et al. reported the outcomes of the high-grade gliomas treated with proton therapy. The treatment was well-tolerated, and the survival rates were comparable to previous photon-based therapy [10]. The short-term toxicities caused by proton therapy were mainly alopecia, headache, or fatigue. These common side-effects following proton therapy were the same as low-grade gliomas or other intracranial tumors [38–40]. In a phase II trial by Brown et al. [12], there was no indication of improved survival outcomes with proton therapy compared to IMRT. But adverse reactions have been slightly reduced by proton therapy. According to dosimetry analysis, proton significantly reduced the minimum, average, and maximum dose for organ-at-risks. The physical properties of the proton beam seem to protect normal structures and reduce toxicity. Previous studies have found that intensity-modulated proton therapy (IMPT) can provide more conformal target coverage than IMRT [19, 41]. Similar outcomes with other malignant tumors found less toxicity after proton therapy than IMRT [42–45]. The different survival outcomes have not been produced by the same radiation dose of the two techniques, so the dose-escalation model for proton therapy will be applied to more prospective research. A promising prospective randomized study (NCT02179086) compared dose-escalated protons to photons for GBM [46] (refer to Table 5).

The current standard of care for high-grade glioma (HGG) patients with photon therapy remains unsatisfactory. The pattern of treatment failure is mainly recurrence in the local region, which is an intractable problem with poor treatment outcomes. Long-term local control for most remains elusive, and local recurrence within 2 cm of the residual cavity is inevitable [26, 47]. McDonald et al. reported that 93% of patients had a central or in-field recurrence after standard care of photon therapy (total dose 60 Gy) plus temozolomide. Novel approaches may increase the target dose intensity while normal brain tissues are spared. Dose-escalation applied for proton boost in previous studies (developed by Fitzek et al. [15]) provided new insights on improving tumor control rates. The escalation of the dose to 90 CGE with a combination of photons and protons boost improved the local control of HGGs. The 2-year OS% and mOS were 34% and 20 months, respectively [15]. The recurrent region is often in the periphery of this 90 CGE volume (frequently observed in regions that received ≤ 60-70CGE). Therefore, the core technical challenge is accompanied by an increase in the target dose, limited by radiation brain necrosis. Although the standard plan was not agreed upon at that time, the innovative dose-escalation referred to some phenomenon in which radiation necrosis survived significantly longer than patients with recurrence (*p* = 0.01). But the patients endured repeated operations and continuing medical treatment owing to radiation necrosis or recurrence. The use of hyper-fractionation theoretically benefits a large total radiation dose without excessive late toxicity. It has been investigated in many clinical trials involving a wide range of tumor types [48, 49]. Mizumoto et al. [17] evaluated the survival of GBM patients after hyper-fractionated concomitant X-ray and proton boost (more than 6 h after conventional radiation therapy). Total doses of 96.6 GyE in 56 fractions can effectively suppress GBM growth. The area irradiated with > 90 GyE had great local control, while local recurrence was observed in areas which received less than 73.5GyE. Although the physical properties of the proton beam allow dose-escalation radiation on target, brain necrosis is inevitable. In this trial, brain necrosis occurs later than tumor recurrence. The radiation necrosis was observed in 6(26%) of 23 patients; moreover, all the patients (100%) experienced brain necrosis, according to Fitzek et al. [15]. But overall survival in patients with brain necrosis was significantly greater than that of those with tumor recurrence. The same findings that patients with necrosis treated by standard therapy followed by necrotomy had improved overall survival compared to those with tumor recurrence are reported by Rusthoven et al. [50]. Radiation necrosis has often been treated by necrotomy, drugs, hyperbaric oxygen therapy, etc. Recently, some researchers have found that bevacizumab is an effective treatment for radiation necrosis [51, 52]. Two patients treated with bevacizumab in hyper-fractioned proton trial showed favorable responses, relieving the clinical symptoms. Overall, high-dose proton therapy (96.6 GyE) is a potential way to improve local control. Moreover, prolonged hospitalization greatly strains medical resources, perhaps making it difficult to apply into practice.

X-radiation followed by proton radiotherapy as a sequential boost is also a treatment of HGGs. A retrospective study was undertaken at Heidelberg Ion-Beam Therapy Center, comparing sequential proton boost after standard chemoradiation with photon-based concurrent radio-chemotherapy to analyze the feasibility and safety [14]. The median PFS and OS in both treatment groups were comparable, and there were no discrepancies in the historical data [10]. In terms of adverse effects, proton boost was at the very least potentially better than typical standard schemes [10]. Compared with the foregoing carbon ion boost from the SPHIC study, the biological benefits of heavy particles appear to be greater than those of proton boost therapy. A prospective CLEOPATRA trial (NCT01165671) is in progress in patients with glioblastoma to compare the impact of a carbon-ion boost with a proton boost using IMRT [53] (refer to Table 5). Proton or carbon-ion boost is given to macroscopic residuals for patients after surgery. Whether this method could maximize efficacy, we look forward to releasing the results of this study online.

In summary, eight clinical trials regarding charged-particle therapy for high-grade gliomas involved carbon-ion boost combined with proton or photon, proton alone, dose-escalation of proton as boost, and proton boost therapy. All the techniques show favorable treatment-response with controllable toxicity. In comparison with IMRT, proton alone therapy might not have good survival outcomes and local tumor control advantages. Although salvage therapies are effective, dose-escalation of proton as boost produced radiation necrosis. In the future, amino acid positron emission tomography (PET) with 11C-methionine (MET) or 18F-fluoroethyltyrosine (FET) could be more widely used for target delineation to improve the accuracy of radiation areas. Proton therapy as sequential boost is inferior to carbon ion boost in terms of biological effects, but more prospective trials are needed to carry it out.

This systematic review summarizes the dosimetry advantages of protons over all types of photon techniques to provide precision options. In addition, the analysis of novel charged particle measures can contribute to practical applications. Three articles were used to obtain the parameters of the target dosimetry between protons and photons. When an intensity-modulated proton beam (known as a pencil beam or active scattering) as a novel mode of proton therapy is applied to these three dosimetry studies, it allows for a high-quality target plan that optimizes dose distributions. However, the evaluation criterion could not reach consistency among these plans. It is now generally accepted that the homogeneity index and conformity index are used to compare target dosimetry [54–58]. Overall, homogeneity was significantly improved with protons, regardless of the tumor treatment plan [59–64]. Similarly, Adeberg et al. [18] discovered that the HI was high for the proton, and other parameters for evaluating CTV coverage were not different, except that Dmin was shown to be higher in proton plans compared to others. All three modalities were comparable to CTV coverage. Considering that dose coverage and distribution were difficult to distinguish particularly when using the same prescribed dose. Dose escalation may be achieved with protons. Another reason for this might be that the radiation therapist tended to spare important OARs in photons if they were in proximity to the target, as depicted by the high CTV Dmin using protons. For other parameters including PTV coverage, R30, or ODV, it was illustrated that proton techniques produced more conformal target doses than photon techniques. Previous studies drew the same conclusions as well [65–68].

Dosimetry assessment of OARs for charged particle therapy is important. Adverse reactions related to the quality of life or neurocognitive function in patients need to be considered, not merely survival rates. The physical properties of charged particles seem to decrease the exposed volume of normal tissue [69–72]. Dose assessment of main intracranial OARs was classified by functions, including sensory (optic nerve, chiasm, lens, etc.), endocrine (hypothalamic-pituitary axis), neurocognition system (hippocampus, SVZ, whole brain). In our analysis of dosimetry data of OARs, the benefits of protons are clear in terms of sparing normal contralateral brain tissues. Proton therapy significantly reduced the contralateral Dmean, Dmax, and integral dose in the optic nerve, brainstem, hippocampus, and other brain regions. Similar studies have also reported that reducing radiation exposure in protons resulted in cerebral OARs, particularly in contralateral non-target normal tissues [70, 73]. Therefore, the decreased risk of radiation-related side effects makes it possible to treat patients with proton beam.

This systematic review has several limitations. First, due to the scarcity of multi-institutional randomized controlled trials (RCTs), all clinical trials involved appear to be of low quality. Furthermore, the small sample size and short-term follow-up period made it difficult to accurately observe the course of treatment outcomes. Second, every concrete charged particle technique applied in clinical studies failed to reach a consistent standard. For example, carbon ion boost used at two different institutes as a treatment measure with various dose fractions or dose intensities is difficult to compare with other techniques. Third, some studies did not adhere to the guidelines for standard treatment, particularly in terms of tumor resection or the administration of chemotherapeutic agents. These factors would an explicit effect on the treatment efficacy. Moreover, more prospective trials regarding dosimetry comparison of charged particles and photon therapy should be taken further into consideration. Finally, the O^6^methylguanine-DNA methyltransferase status and IDH mutation were not assessed in the majority of research. Alterations to these molecular levels are needed. Three prospective trials (NCT01358058, NCT03180502, NCT02797366, sponsored by NRG Oncology, Sweden and the United States) are conducted regarding proton therapy treating WHO grade 3 glioma with different molecular pathology (IDH1 mutation or 1p/19q codeletion) (see Table 5). This will bring us new ideas to guide different types of patients to benefit from treatment (Table 6).

**Table 6:** Dosimetry comparison study

Authors (year)	Tumor histology	Patient number	Technique types	Target prescription dose and a fraction (Gy/RBE/CGE)	Comparative parameters of treatment plans	Evaluation parameters for OARs	Conclusions
Rosenschöld et al. [19]	High-grade glioma	15	Dosimetry comparison between IMRT, VMAT and IMPT	IMRT/VAMT (PTV:60.0 Gy/30Fx) IMPT(PTV:60GyE/30Fx)	PTV dose conformity: Absorbed dose standard deviation for PTV	Dmax, Dmin, and Dmean evaluated for the normal brain (hippocampus, eyes, optic chiasm, fiber objects, brainstem)	The IMPT produced the most conform plans among other techniques
Adeberg et al. [18]	High-grade glioma	12	Dosimetry comparison between 3D-CRT, VMAT and IMPT	IMPT (Median dose of 60.0 GyE (range 56.0–60.0GyE)/30Fx) IMRT/VAMT(PTV:60.0 Gy/30Fx)	CTV coverage: V_90%_, V_95%_, V_100%_, Dmax, Dmean, Dmin, HI, IC, ID	Dmax and Dmean evaluated for the normal brain (Chiasm, Optic nerve, Brainstem, Lens, Pituitary gland axis, Hippocampus, SVZ, Whole brain)	IMPT can serve as a valuable alternative to photon therapy for HGG
Holm et al. [20]	High-grade glioma	5	Dosimetry comparison between IMRT, VMAT and IMPT	Standard IMRT plan: (PTV_boost_:60.0 Gy/30Fx); Dose escalation plan: Dmean to PTV_boost_ (77.1 Gy for IMRT, 79.2 Gy for VMAT and 85.1GyE for IMPT); Dmax (81.3 Gy, 86.9 Gy and 89.3 GyE, respectively)	Dose-escalation plan: PTV_boost_ (Dmax,Dmean Gy/GyE); ODV; R30	Dmean evaluated for the normal brain (Temporal lobe, Spinal cord, Optic nerve, Lens, Hippocampus, Eyes, Cochlea, Brainstem, Chiasm)	IMPT being the most favorable technique among others

*OARs* organ at risk; *SVZ* Subventricular zone; *IMRT* intensity modulated radiation therapy; *VMAT* volumetric arc therapy; *IMPT* intensity modulated proton therapy; *HI* homogeneity index; *IC* inhomogeneity coefficient; *ID* Integral dose; *ODV* overdose volume; *R30* the ratio of the 30 Gy isodose curve and the boost volume; *V*_90%_ percentage of CTV receiving a minimum of 90% of the prescribed dose; *V*_95%_: percentage of CTV receiving a minimum of 95% of the prescribed dose; *V*_100%_: percentage of CTV receiving a minimum of 100% of the prescribed dose

In conclusion, the use of charged particle radiotherapy seems to be effective with acceptable adverse effects when used either alone or as a boost. To date, no adequate evidence validates which protocol is much more effective. However, survival outcomes show that carbon ion boost is seemingly superior to proton boost concerning biological effects. In terms of toxicity, the side effects of carbon ion and proton therapy could be accepted. Still, the way of dose-escalation as proton boost occurs, brain necrosis should not be overlooked, despite the survival rates improving. The proton beam could provide good target coverage, and it seems to reduce dose exposure to contralateral OARs significantly. This can potentially reduce the dose- and volume-related side effects of treatment, such as neurocognitive impairment, in long-term survivors. Furthermore, high-quality RCTs should be conducted in the future. Moreover, systemic therapy options combined with charged particles are necessary.

## Abbreviations

RBE: Relative biological effectiveness
PROSPERO: Prospective register of systematic reviews
HGGs: High-grade gliomas
GBM: Glioblastoma multiforme
AA: Anaplastic astrocytoma
OS: Overall survival
PFS: Progression-free survival
ACNU: Nimustine hydrochloride
GyE: Gray equivalent
IMRT: Intensity-modulated radiation
PTV: Plan target volume
GTV: Gross target volume
CGE: Cobalt gray equivalent
VMAT: Volumetric modulated arc therapy
